# The Effect of Mushroom Dietary Fiber on the Gut Microbiota and Related Health Benefits: A Review

**DOI:** 10.3390/jof9101028

**Published:** 2023-10-19

**Authors:** Changxia Yu, Qin Dong, Mingjie Chen, Ruihua Zhao, Lei Zha, Yan Zhao, Mengke Zhang, Baosheng Zhang, Aimin Ma

**Affiliations:** 1 Institute of Edible Fungi, Shanghai Academy of Agricultural Sciences, Shanghai 201403, China; yuchangxia@saas.sh.cn (C.Y.); maomao88719@163.com (Q.D.); mjfungi@126.com (M.C.); zhalei@saas.sh.cn (L.Z.); zmk1845342941@163.com (M.Z.); bsjiayou0912@163.com (B.Z.); 2 School of Life Sciences, Yan’an University, Yan’an 716000, China; zhaohua506@sohu.com; 3 College of Food Science and Technology, Huazhong Agricultural University, Wuhan 430070, China

**Keywords:** dietary fiber, mushroom, gut microbiota, beneficial effects, short-chain fatty acids

## Abstract

Mushroom dietary fiber is a type of bioactive macromolecule derived from the mycelia, fruiting bodies, or sclerotia of edible or medicinal fungi. The use of mushroom dietary fiber as a prebiotic has recently gained significant attention for providing health benefits to the host by promoting the growth of beneficial microorganisms; therefore, mushroom dietary fiber has promising prospects for application in the functional food industry and in drug development. This review summarizes methods for the preparation and modification of mushroom dietary fiber, its degradation and metabolism in the intestine, its impact on the gut microbiota community, and the generation of short-chain fatty acids (SCFAs); this review also systematically summarizes the beneficial effects of mushroom dietary fiber on host health. Overall, this review aims to provide theoretical guidance and a fresh perspective for the prebiotic application of mushroom dietary fiber in the development of new functional foods and drugs.

## 1. Introduction

Microorganisms are extensively distributed in all parts of the human body. Some microorganisms are synergistically associated with the human body and are thus called symbiotic microorganisms [1]. These microorganisms are mainly distributed on the skin surface, oral cavity, digestive system, respiratory system, and urogenital system [2], with 95% of them inhabiting the human intestinal tract [3]. The gut microbiota comprises up to trillions of microorganisms (10 times the number of human cells), with as many as 1000 species included. On average, each human has approximately 160 microbial species in the intestinal tract [4,5], with 90% of species belonging to the phyla *Firmicutes* and *Bacteroidetes*, followed by *Proteobacteria*, *Actinobacteria*, and *Fusobacteria* [6]. The gut microbiota encodes more than three million genes, 150 times more than those encoded by the human genome [7]. Therefore, the gut microbiota is also known as the “second human genome” [8]. Numerous studies have recently reported that the gut microbiota is closely involved in energy homeostasis [9], immune system regulation [10], metabolism [11], and other physiological processes in the host. Thus, the gut microbiota is also termed the “hidden metabolic organ” [12]. The human gut microbiota is a complex, interactive, and dynamically balanced ecosystem. Dietary changes, diseases, drugs, and other factors cause disturbances and changes in the composition of the gut microbiota, sometimes resulting in dysbiosis [13,14]. Numerous studies have shown that dysbiosis of the gut microbiota is correlated with the occurrence of several chronic diseases, such as obesity [15], diabetes [16], liver disease [17], inflammatory bowel disease [18], and cancer [19].

Mushrooms are valuable and healthy and they have a long history of consumption and have increased in popularity in recent years worldwide [20]. They are mainly composed of basidiomycete or ascomycete fungi and are prized for their nutritional and medicinal properties [21]. Bioactive compounds such as proteins, vitamins, minerals, dietary fibers, and trace elements from different mushroom varieties have been demonstrated to have high nutritional value [22] and enhance human health by promoting antioxidant, antimicrobial, anti-inflammatory, anticancer, antitumor, and immunostimulatory effects [23]. Mushrooms are popular among consumers as both a medicine and food. Peptides, lectins, ergosterol, terpenoids, phenols, and other biologically active compounds have been isolated and identified from various mushrooms [24]. However, mushrooms have relatively low levels of these active ingredients. Dietary fiber (DF), known as the “seventh nutrient” [25], positively affects blood sugar, blood pressure, lipid metabolism, and inflammation. The total DF content in the sclerotia of some mushrooms can exceed 80%. For example, the total DFs extracted from the sclerotia of *Pleurotus tuber regium*, *Polyporus rhinocerus*, and *Wolfifiporia cocos* were 81.7–96.3% of the total content [26,27]. A high fiber content raises the new possibility of using mushrooms as functional foods. Several studies have reported that DFs from mushrooms such as *Lentinula edodes* and *Hericium erinaceus* can change the gut microbiota, and therefore, DFs from mushrooms have attracted increasing attention [28,29,30]. DFs from mushrooms act as prebiotics. The selective growth of particular microorganisms in the intestine can stimulate the growth of beneficial microorganisms and inhibit the proliferation of pathogens, thus altering the gut microbiota to improve health [31]. This paper reviews the methods of preparing and modifying mushroom-derived DFs and their regulatory effects on the gut microbiota. In addition, we discuss how this modulation of the gut microbiota benefits the host. Our findings will provide theoretical guidance and insight for researchers seeking to develop new functional foods or drugs using mushroom-derived DFs.

A comprehensive literature search was conducted for studies published by 2023 by using the keywords “dietary fiber, mushroom, gut microbiota” on PubMed, Web of Science, cross ref, Elsevier, Springer Link, Google Scholar, and Scopus. The retrieved articles were characterized based on the methods of preparation and modification, degradation and metabolism in the intestine, and the beneficial effects of mushroom dietary fiber. The content of the review has been arranged and presented in specific sections.

## 2. Composition of DF from Mushrooms

Structurally, DF is a carbohydrate polymer with a polymerization degree of at least 10 and is typically associated with health benefits. DF cannot be digested or absorbed in the small intestine. DF can be naturally obtained from raw food materials or synthesized through physical, enzymatic, or chemical methods [32]. Based on its dissolution characteristics, DF is classified as soluble dietary fiber (SDF) and insoluble dietary fiber (IDF) [33]. DFs from different sources exhibit different structures, chemical compositions, and physicochemical properties. Moreover, DF has various nutritional and physiological benefits. Compared with DFs from traditional sources, such as grains, vegetables, and fruits, the potential of DFs from mushrooms has not been fully realized [34,35]. In fact, mushrooms are rich in new types of DFs that are suitable for various members of the population, including children and those with diabetes. Thus, mushroom DFs have varied beneficial effects on human health [36]. DFs in mushrooms mainly include chitin (a straight-chain (1→4)-β-linked polymer of N-acetyl-glucosamine), β-glucan, and hemicellulose [37]. Among them, β-glucan is recognized as one of the most important components and is primarily linked by the mixed linkage of the β-1,4 and β-1,6 glycosidic bond, as well as the single linkage of β-1,3; β-1,4; and β-1,6 [38]. β-glucan is present in both SDF and IDF in mushrooms. However, its proportion in SDF and IDF considerably varies based on mushroom genera. In general, the proportion of β-glucan is higher in IDF [39].

## 3. Methods of the Preparation and Modification of DFs from Mushrooms

### 3.1. The Preparation of DFs from Mushrooms

**Figure 1:**
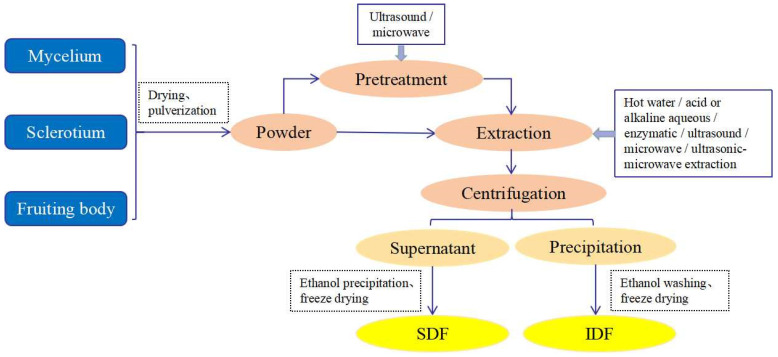
Schematic showing an overview of mushroom DF extraction.

Using the extraction process shown in Figure 1, DFs from mushrooms are separated from the fruiting bodies, mycelia, or sclerotia [26,31]. As the first step of extraction, the dried mushroom fruiting bodies, mycelia, or sclerotia are ground into powder. The extraction is performed using ultrasonic or microwave treatment, or the powder is directly extracted with hot water, acid or alkaline aqueous solution, enzyme, ultrasonic waves, and other methods. Then, DFs are further isolated through centrifugation, ethanol precipitation, and freeze drying [40,41,42]. The type of extraction method affects the physicochemical properties and potential bioactivity of DFs; thus, the extraction method and extraction parameters, including solvent type, extraction temperature, extraction time, liquid–solid ratio, and equipment power, should be considered before DF extraction [43]. The methods for extracting DF from mushrooms include chemical, physical, enzyme, and microbial methods or a combination of these. Among them, alkali and enzymatic extraction methods are the most frequently used. The alkaline aqueous method is more widely used than the enzymatic method because it involves a simple protocol, has a low cost, and can be easily controlled [44,45]. The comparison of different methods and technical strategies used to extract DFs from mushrooms is shown in Table 1.

### 3.2. Methods of Modification of DF from Mushrooms

There is a considerable difference in the DF content of different mushrooms. According to a paper by Cheung (2008) [54], the SDF content of some mushrooms is 0.50–4.42%, while the IDF content ranges from 23.6 to 43.1%. SDF has many crucial physiological functions because of its good gelling, water absorption, swelling, and fermentability properties [55,56,57]. The SDF content in high-quality DF should be more than 10% [58]. Therefore, increasing the content of SDF in mushrooms is a goal of modification. The currently used DF modification methods are divided into the following four main types: physical methods, chemical methods, biological methods, and combination methods. Treatment with different modification methods causes corresponding changes in the composition and structural characteristics of DFs, thereby affecting the physicochemical properties of DFs, including their oil holding capacity (OHC) and adsorption capacities [59]. The conditions, properties, and yield changes in mushroom DF after the application of different modification methods are shown in Table 2.

#### 3.2.1. Physical Modification

The methods of physical modification involve modification through the destruction of the glycosidic bonds of DFs by applying external high temperature, high pressure, instantaneous decompression, explosion, high-speed impact, or shearing. Some examples of physical modification methods are steam treatment (SP) [60], high-pressure homogenization (HPH) [61], dynamic high-pressure microfluidization (DHPM) [62], ultrasonic comminution (UC) [63], high hydrostatic pressure (HHP) [63], extrusion [64], ultrasound [65], microwave [66], and cavitation jet processing [67]. The physical modification methods produce good results, have high production efficiency, and generate no chemical reagent residue; therefore, these methods are widely used but require a large investment in equipment.

#### 3.2.2. Chemical Modification

In the methods for chemical modification, the structure and functional properties of DFs are modified through chemical reactions. Some examples of chemical modification methods include treatments with alkaline hydrogen peroxide [68], acid carboxymethylation [69], and hydroxypropylation [70]. DF modification using chemical methods is associated with a low cost but can alter the structural and functional properties of DFs. Furthermore, these methods may be associated with problems related to reagent residue.

#### 3.2.3. Biological Modification

In the methods of biological modification, specific enzymes or microorganisms are utilized for the enzymolysis or fermentation of raw materials to modify DFs. Biological modification methods can be enzymatic [71] or based on microbial fermentation methods [72] and require mild treatment conditions that reduce DF loss; however, the production efficiency of these techniques is low. The high cost of the enzymatic method and the development of highly active strains involved in fermentation currently prevent the wider adoption of these techniques.

#### 3.2.4. Combination Modification

The combination method refers to DF modification with two or more of the aforementioned methods [73]. The combination modification method can effectively compensate for the shortcomings of a single method and is likely to become the focus of future research.

**Table 2:** Modification methods, properties, and yield changes of mushroom DFs.

Modification Methods		Material	DFs	Modification Conditions	Property Changes	Reference
Physical modification method	High-pressure homogenization	*Flammulina* *velutiper*	IDF	0, 10, 30, and 50 cycles at 700 bar	WHC ↑, interfacial properties ↑, particle size ↓, emulsification Performance ↑	[61]
	Extrusion	*Lentinula edodes* residues	DF	130 °C, moisture content 40%, 125 r/min	SDF ↑, OHC ↑, GAC ↑, glucose retardation and bile acid retardation index ↑	[64]
	High-temperature cooking	*Flammulina* *velutiper*	DF	Liquid-to-material ratio 30:1, 125 °C, 50 min	SDF ↑, improves the physiological indices in obese mice	[74]
	High-pressure processing	*Agrocybe chaxingu*	DF	400 MPa, 25 °C, 15 min	SDF ↑, polysaccharide solubility ↑, lower viscosity and greater fluidity	[75]
Chemical modification method	Alkaline	*Lentinus edodes* stem	DF	13% NaOH, 80% ethanol, alkalization 120 min; 10% C2H2ClNaO2, 50 °C, etherification 3.5 h	WHC ↑, SC ↓, OHC ↑	[76]
Biological modification method	Enzymatic	*Lentinus edodes*	DF	1.5% cellulase, solid–liquid ratio 1:25, 50 °C, pH 5.5, 120 min	SC ↑, WHC ↑, OHC ↑, cation exchange capacity ↑, GAC ↑	[71]
	Fermentation	*Lentinus edodes* stem	IDFSDF	Material-liquid 1:10 g/mL, 6% *Aspergillus niger*, 28 °C, 2 d	IDF: WHC ↑, OHC ↑, SC ↑; SDF: WHC ↑, OHC ↑, SC ↑	[72]
Combined modification method	Enzymatic-chemical	*Auricularia polytricha*	DF	0.4% α-amylase 1.0% protamex, 66 °C, liquid material ratio 41 mL/g	SC ↑, WHC ↑, FAC ↑, GAC ↑, high constipation-relieving activity	[77]
	Ultrasound-microwave-assisted enzymatic method	*Hericium* *Erinaceus* residue	DF	3% celluloses, ultrasound (1.5 W/mL), 50 °C, 75 min, boiled to stop the enzyme	SDF ↑, particle size ↓, adsorption capacity ↑, better blood lipid-lowering effect in vitro	[78]

Note: WHC: water holding capacity; OHC: oil holding capacity; GAC: glucose adsorption capacity; SC: swelling capacity; FAC: fat adsorption capacity; ↑: increase; ↓: decrease.

## 4. Interaction between DFs and the Gut Microbiota

**Figure 2:**
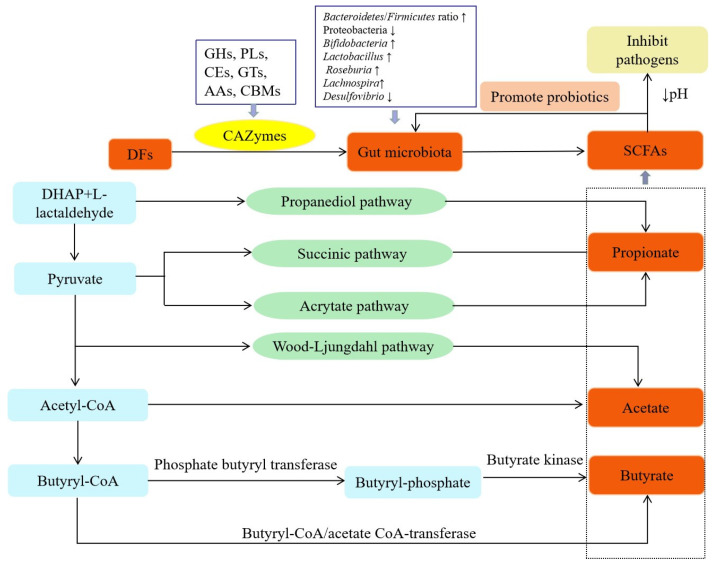
The effects of DFs on gut microbiota metabolism. Note: ↑: increase; ↓: decrease.

DFs can be utilized by the gut microbiota. DFs exert beneficial effects on the host mainly by fermentation and the production of metabolites. The effect of DFs on the gut microbiota is summarized in Figure 2.

### 4.1. The Role of the Gut Microbiota in DF Metabolism

The gut microbiota affects the digestion, immunity, and nervous systems of human hosts by metabolizing carbohydrates, protein, fat, and other substances in the body [79]. The human genome cannot encode a sufficient amount of carbohydrate-active enzymes (CAZymes) for different glycosidic bonds [80]. Therefore, only some simple carbohydrates are digested in humans, and the remaining complex carbohydrates, including DFs, are transported to the large intestine for use by the gut microbiota [81]. Numerous CAZymes are produced by the gut microbiota and are involved in regulating the metabolism and utilization of carbohydrates such as DFs by the gut microbiota, thereby aiding the human digestive system in carbohydrate degradation and producing absorbable short-chain fatty acids (SCFAs) and other metabolites [82]. The gut microbiota can produce various CAZymes needed for DF degradation. According to differences in the similarity of amino acid sequences, protein structure, and catalytic function, CAZymes are categorized into five types of catalytic enzymes and the noncatalytic carbohydrate-binding module (CBM) [83]. The catalytic CAZymes include glycoside hydrolases (GHs), polysaccharide lyases (PLs), carbohydrate esterases (CEs), glycosyltransferases (GTs), and auxiliary activities (AAs). GHs degrade glycosidic bonds between two or more carbohydrates and those between carbohydrates and noncarbohydrates [84]. PLs degrade the long uronic-acid-containing polysaccharide chains through the β-elimination mechanism [85]. CEs remove ester groups in carbohydrates and participate in reactions involving side-chain degradation [86]. GTs catalyze the transfer of glycosyl groups from activated donor molecules to specific receptor molecules to form glycosidic bonds [87].

During DF degradation, collaboration between various CAZymes is necessary. For example, Ndeh et al. confirmed that the synergism of GHs, PLs, CEs, and other enzymes is needed for rhamnonic acid II degradation by *Bacteroides polymorphus* [88]. Some intestinal microorganisms can use numerous carbohydrates with different structures, whereas others can use only a small amount of carbohydrates [89]. According to Zhang et al., on average, *Bacteroidetes* encode four times more CAZyme genes than *Firmicutes* [90]. Approximately 81% of GHs and PLs in *Bacteroidetes* have signal sequences, whereas only 19% of GHs and PLs in *Firmicutes* have signal sequences [91]. Therefore, *Bacteroides* are better able to metabolize carbohydrates. In addition, intestinal microorganisms can degrade complex carbohydrates through cooperation. Degrading all carbohydrates in the intestinal tract is difficult for only one type of intestinal microorganism [92]. Thus, the short-chain primary products produced by some microorganisms that degrade complex carbohydrates can be transferred to other microorganisms for further degradation [93]. For example, *Eubacterium rectale* only decomposes the gum aldose side-chain of arabinoxylan, while *Bifidobacterium longum* further metabolizes these primary products to form monosaccharides, which are later consumed by *E. rectale* [94].

### 4.2. Effect of DFs on the Composition of the Gut Microbiota

Many factors affect the composition and function of the gut microbiota, including the host’s age and sex, genetic background, physiological status, living environment, diet habits, and drug treatment [95,96]. Of them, diet is considered among the most important factors because it significantly affects the composition, diversity, and abundance of the gut microbiota [97]. DFs are considered a nutritional source for the gut microbiota and play a major role in host health. Decreases in DF intake are associated with decreasing gut microbiota abundance, and vice versa [89,98,99]. Many recent in vivo and in vitro experimental studies have shown that DFs in mushrooms have a regulatory effect on the gut microbiota (Table 3). Mitsou et al. [100] suggested that mushrooms rich in β-glucans may exert beneficial in vitro effects on gut microbiota and/or SCFA production in elderly subjects. Zhang et al. [50] found that *Coprinus comatus* DFs regulated the gut microbiota composition by increasing the abundance of *Bacteroides* and *Bifidobacterium* and reducing the *Firmicutes/Bacteroides* ratio during an in vitro fermentation test; Zhao et al. [101] also confirmed that *Flammulina velutipes* DFs reduce the *Firmicutes/Bacteroidetes* ratio; and through in vitro experiments, Han et al. [53] demonstrated that *Pleurotus eryngii* DFs regulate the gut microbiota composition in mice fed a high-fat diet (HFD) by increasing the abundance of beneficial microorganisms such as *Metallobacterium* and *Lactobacillus* and reducing the abundance of harmful microorganisms such as *unidentified_Lachnospiraceae* and *Helicobacter*. All these results indicate that DFs from varying sources have a range of effects on the gut microbiota composition and abundance in vivo and in vitro, which may be related to differences in structure and glycosidic bond types [102,103].

The function of DFs in regulating gut microbial diversity and composition has become a research hot spot. According to previous studies, DFs can be used as a substrate for intestinal microorganism CAZymes, and SCFAs produced through fermentation decrease the intestinal pH, thereby promoting the growth of beneficial microorganisms and inhibiting the growth of pathogenic microorganisms; this further affects the gut microbiota composition and the balance of microbial metabolites [104]. For example, during in vitro fermentation, DFs from *Agaricus bisporus* reduced the pH from 6.93 to 4.48 [105]. Similarly, DFs from *Lentinus edodes* and *Ganoderma atram* significantly reduced the pH when fermented [106,107]. On the other hand, pH significantly affects the abundance and diversity of the gut microbiota and enzyme activity [108,109]. pH may further affect the metabolism of the gut microbiota. For example, *Bacteroides* have a stronger adaptability at pH 6.7 than at pH 5.5, whereas *Firmicutes* have a stronger adaptability at pH 5.5 [110]. Moreover, the gut microbiota forms an interdependent community, wherein some intestinal microorganisms induce the growth of other microorganisms through cross-feeding behavior, thereby enriching the diversity and maintaining the stability of the gut microbiota [111,112]. For instance, *Eubacterium hallii* utilizes the products of 1,2-propanediol from the fermentation of rhamnose by *Blautia* spp. [113]. *Bifidobacterium* sp. can degrade starch or fructooligosaccharides, which can stimulate the growth of species in coculture that cannot degrade these complex substrates [114,115].

**Table 3:** Effects of mushroom DF on the gut microbiota and SCFAs.

DF Source	Model	Gut Microbiota Regulation	SCFA Generation	Effect on Host	Reference
*Pleurotus eryngii*	HFD-induced obese rat	The relative abundances of *Roseburia* and *Lactobacillus* ↓, the relative abundances of *Anaerostipes*, *Clostridium* and *Lactococcus* ↑.	Increased the concentrations of total SCFAs.	Reduced BW gain, adipose tissue weight, FBG level; the expression of *FASN* and *ACC*.	[116]
*Pleurotus eryngii*	HFD-fed mice	The relative abundances of *Methylobacterium* and *Lactobacillus* ↑, the relative abundances of *unidentified_Lachnospiraceae* and *Helicobacter* ↓.	Increased the content of SCFAs, including acetic acid, propionic acid, and butyric acid.	Decreased the weight, promoted the proliferation of beneficial bacteria, reduced the risks of many chronic diseases.	[53]
*Agaricus blazei* Murrill	Hyperlipidemia rats	The ratio of *Firmicutes*/*Bacteroidetes* ↓; the abundance of *Peptostreptococcaceae*, *Erysipelaceae,* and *Clostridium* ↑.	Nm	Regulated dyslipidemia in rats with hyperlipidemia possibly by regulating imbalance in the intestinal microflora.	[117]
*Hericium caput-medusae*	One-day-old Arbor Acres male broilers	The count of *Lactobacilli* and *Bifidobacteria* ↑, the count of acecum *Escherichia coli* ↓.	Increased the concentration of propionic acid.	Decreased cholesterol content in broiler chickens.	[118]
*Flammulina velutipes*	Male C57BL/6 J mice	The relative abundance of some beneficial bacteria ↑, such as *Akkermansia* and Prevotellaceae UCG-001; the relative abundance of some harmful bacteria ↓, such as *Lachnospiraceae* NK4A136 group and *Desulfovibrio.*	Nm	Reduced the weight gain, triglycerides and total cholesterol, low-density lipoprotein cholesterol; increased the activity of enzymes related to scavenging ability of oxygen free radicals.	[119]
*Flammulina velutipes*	Mice	The relative abundance of *Firmicutes* ↓, the relative abundance of *Bacteroidetes* ↑; the ratio of *Firmicutes*/*Bacteroidetes* ↓.	Increased the concentrations of total SCFAs, acetic acid, propionic acid, and n-butyric acid.	Suppressed obesity and immune regulation.	[101]
*Ganoderma lucidum*	C57BL/6NCrlBltw genetic lineage mice	The ratio of *Firmicutes*/*Bacteroidetes*, *Proteobacteria* ↓.	Nm	Reduced body weight gain, chronic inflammation, and insulin resistance in obese individuals.	[120]
*Poria cocos*	C57BL/6J mice	The relative abundance of *Lachnospiracea*, *Clostridium* ↑.	Increased butyrate levels.	Activated the intestinal PPAR-γ pathway, modulated gut microbiota to improve hyperglycemia and hyperlipidemia.	[121]
*Agaricus bisporus*	Human	The relative abundance of *Firmicutes* ↑, the relative abundance of *Bacteroidetes* ↓.	Increased the concentrations of acetic acid and propionic acid.	Increased the relative abundance of beneficial bacteria, exhibited an effective prebiotic regulation function on human gut microbiota.	[105]
*Cordyceps militaris*	Liver and kidney injury induced by lead acetate in mice	The relative abundance of *Clostridium* and *Bacteroidetes* ↑, the relative abundance of *Firmicutes* ↓.	Nm	Reduced the Pb^2+^ content and organ index of liver and kidney in mice, had a protective effect on organs against damage in mice.	[122]
*Pleurotus eryngii*	C57BL/6 male mice	The relative abundances of *Firmicutes* ↓, *Bacteroidetes* ↑	Increased the concentrations of Acetate and Propionate.	Regulated the host immune function effectively.	[123]
*Ganoderma lucidum*	Chronic pancreatitis mice	The relative abundance of *Bacteroidetes* ↓ and that of *Firmicutes* ↑; at the genus level, the relative abundance of beneficial bacteria such as *Lactobacillus*, *Roseburia,* and *Lachnospira* ↑.	Nm	Indicated beneficial effects on pancreas fibrosis, and impeded an inflammatory response.	[124]
*Dictyophora indusiata*	Antibiotic-induced intestinal microflora disorder in mice	Beneficial bacteria ↑, including *Lactobacilli* and *Ruminococcaceae*; harmful bacteria ↓, such as *Enterococcus*, *Bacteroides*, and *Proteobacteria.*	Nm	Enhanced the restoration of gut microbiota and gut barrier integrity, reduce the inflammation and endotoxin levels in mice.	[125]
*Coprinus comatus*	Human	The relative abundances of *Bacteroides* and *Bifidobacterium* ↑, the ratio of *Firmicutes*/*Bacteroidetes* ↓.	Increased the production of propionic acid and butyric acid.	Demonstrated potential prebiotic effects.	[50]
*Ganoderma lucidum*	C57BL/6J mice	The relative abundances of Actinobacteria at the family level, and *Leuconostoc*, *Lactobacillus* spp. ↑.	Nm	Improved low-grade chronic inflammation, ectopic lipid accumulation, and systemic insulin sensitivity.	[126]
*Hericium erinaceus*	Mice	The relative abundance of *Lachnospiraceae* and *Akkermansiaceae* ↑, the relative abundance of *Rikenellaceae* and *Bacteroidaceae* ↓.	Nm	Promoted the production of NO, IL-6, IL-10, INF-γ, and TNF-α.	[127]
*Auricularia auricular*	ICR mice	The ratio of *Firmicutes*/*Bacteroidetes* ↓, the relative abundance of *Porphyromoadaceae* and *Bacteroidaceae* ↑.	Increased the concentration of total SCFAs and propanoic acid.	Increased microbial community diversity, and increased the immunoglobulin levels in mouse serum.	[128]
*Ganoderma lucidum*	DSS-induced colitis male Wistar rats	The relative abundance of *Firmicutes*, *Paraprevotella*, etc. ↑, the relative abundance of *Proteobacteria*, *Escherichia*, etc. ↓.	Increased total SCFAs, acetic acid, propionic acid, and butyric acid.	Enhanced the immunity and reduced inflammatory response and colonic cancer risk.	[129]
*Ganoderma lucidum*	BALB/C mice	The ratio of *Firmicutes*/*Bacteroidetes* ↓, the relative abundance of *Alistipes* ↑.	Nm	Demonstrated tumor-suppressing activity in mice.	[130]
*Ganoderma lucidum*	BALB/c mice	The relative abundance of *Oscillospira* and unknown genus of *Desulfovibrionaceae* ↓.	Nm	Prevented colon from shortening and reduced mortality by 30% of mortality in CRC mice.	[131]

Note: HFD: high-fat diet; BW: body weight; FBG: fasting blood glucose; FASN: fatty acid synthase; ACC: acetyl-CoA carboxylase; CRC: colorectal cancer; ↑: increase; ↓: decrease.

### 4.3. Effect of DFs on SCFA Production

The fermentation of DF by the gut microbiota yields SCFAs such as acetate, propionate, and butyrate and gases such as CH_4_, H_2_, and CO_2_. As the energy source for colonic epithelial cells, SCFAs play a vital role in cell renewal and recovery. SCFAs can also affect the intestinal mucosal barrier and play a critical role in the health of the intestine [132]. Acetate is the most abundant intestinal SCFA. Intestinal anaerobic microorganisms produce acetate to metabolize pyruvate through acetyl coenzyme A or the Wood Ljungdahl pathway [133]. Propionate is formed by converting succinic acid into methylmalonyl coenzyme A through the succinic acid pathway [134]. Alternatively, propionic acid can be synthesized through the acrylate pathway using acrylic acid and lactic acid as precursors. Additionally, propionic acid can be synthesized through the propanediol pathway, with deoxyhexose (such as trehalose and rhamnose) used as the substrate [135]. Butyrate is reduced to butyryl coenzyme A after condensation with two acetyl coenzyme A molecules. Butyryl coenzyme A is then converted to butyric acid (classical way) through phosphate butyryl transferase and butyrate kinase [136] or through the butyryl coenzyme A/acetic acid coenzyme A transferase pathway [137,138].

SCFAs produced during microbial fermentation participate in metabolism related to different human organs. SCFAs can be quickly absorbed and used by colon cells, transported to the liver through the portal vein system, or enter the circulatory system. Only 5–10% of SCFAs are excreted through feces [139]. SCFAs, especially butyrate, serve as histone deacetylase (HDAC) inhibitors and bind to SCFA receptors to regulate cell proliferation, apoptosis, and differentiation by inhibiting HDACs and altering the expression of functional genes, thereby affecting intestinal function [140]. SCFAs inhibit HDAC and promote the proliferation of macrophages and other cells, the expression of receptors such as Toll-like receptor 4 (TLR4), and the release of anti-inflammatory factors such as interleukin-10 (IL-10). They also inhibit the expression of nuclear factor-κB (NF-κB), tumor necrosis factor (TNF), IL-8, and other cytokines [141,142]. They also combine with different receptors to perform various functions. For instance, butyric acid can induce the arrest of the human colon cancer cell cycle by upregulating the expression of cell cycle regulators and binding to G-protein-coupled receptor 109A (GPR109A) [143]. SCFAs can activate GPR41 and GPR43 and stimulate the secretion of intestinal hormones such as glucagon-like peptide-1 (GLP-1) and peptide tyrosine tyrosine (PYY) in the colon. GLP-1 promotes the body’s secretion of insulin, reduces the secretion of glucagon, and enhances the body’s sensitivity to insulin. PYY can regulate intestinal motility, slow gastric emptying, induce a sense of fullness, and reduce food intake [144]. SCFAs have also been shown to help maintain the integrity of the intestinal mucosal barrier and regulate intestinal motility and the immune response [145,146].

DFs from different mushrooms may affect SCFA production (Table 3). For example, *P. eryngii* DFs increased acetic acid and propionic acid concentrations [123], while *F. velutipes* DFs increased the concentration of total SCFAs, acetic acid, propionic acid, and butyric acid [101]. Moreover, the species and abundance of gut microbiota, substrate source, substrate utilization rate, host genotype, and intestinal transport are factors that influence SCFA production [139].

## 5. Health Benefits of DFs

**Figure 3:**
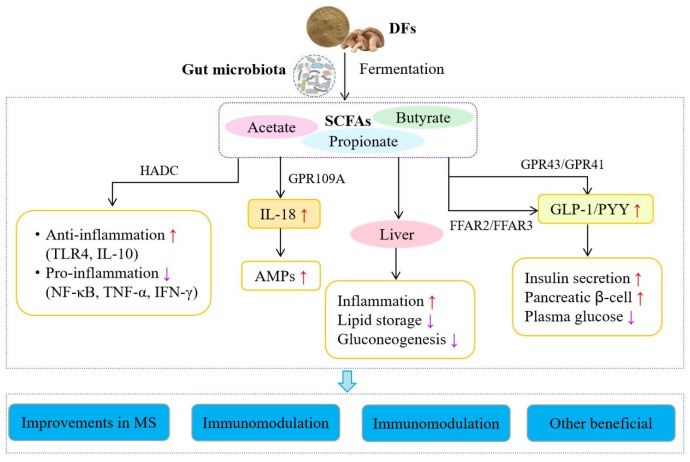
DFs improve host health after oral administration. Note: HADC: histone deacetylase; TLR4: toll-like receptor-4; IL-10: interleukin-10; NF-κB: nuclear factor-κB; TNF-α: tumor necrosis factor-α; IFN-γ: interferon-γ; GPR109A: G-protein-coupled receptor 109A; AMPs: antimicrobial peptides; FFAR2: free fatty acid receptor 2; GLP-1: glucagon-like peptide-1; PYY: peptide YY; MS: metabolic syndrome; ↑: increase; ↓: decrease.

The gut microbiota, as the core microecological system in the human intestinal tract, helps maintain the normal physiological function of the human body by preventing the invasion of various viral antigens. DFs are fermented by intestinal microorganisms to yield SCFAs, which can improve host health and have many beneficial effects in the human body (Figure 3).

### 5.1. Improving Metabolic Syndromes

Metabolic syndrome (MS) is a dysbiosis of physiological metabolism caused by insulin resistance. MS manifests as a pathological state of metabolic disorders related to nutrition, including hyperglycemia, dyslipidemia, central obesity, and hypertension. An increasing amount of evidence indicates that the etiology of MS is associated with dysbiosis of the gut microbiota [147,148,149]. The HFD-induced dysbiosis of the gut microbiota may disrupt intestinal barrier function and increase endotoxin levels in the circulatory system. This leads to metabolic endotoxemia and induces MSs, such as insulin resistance, obesity, and even diabetes [150]. By generating enzymes such as CAZymes and proteases, the gut microbiota promotes the digestion of carbohydrates, such as DFs, and produces metabolites such as SCFAs that can be absorbed and used by the body. Studies have shown that changes in the gut microbiota and SCFAs are associated with the development of metabolic diseases [151]. According to experimental evidence, an increase in *Firmicutes* and a decrease in *Bacteroides* have been observed in obese individuals and mouse models [152]. Indeed, DFs from mushrooms can effectively improve diet-induced MSs in mice and rats (Table 3). For example, *P. eryngii* DFs reduced LDL cholesterol levels and body weight in HFD-fed mice by altering the abundance of SCFA-producing gut microbiota [116]. *G. lucidum* DFs reverse the HFD-induced dysbiosis of gut microbiota by reducing the proportion of *Firmicutes/Bacteroides* and the *Proteobacteria* level. They also maintain the integrity of the intestinal barrier and reduce metabolic endotoxemia [120]. *F. velutipes* DFs can alleviate lipid metabolism dysbiosis in obese mice by regulating the intestinal-flora-mediated AMPK signaling pathway [119]. In addition, other types of DFs from mushrooms can play a positive role in MSs by regulating the gut microbiota composition (Table 3). Thus, mushroom DFs appear to play a positive role in regulating dysbiosis in the intestine that is induced by metabolic disturbances and maintaining the integrity of the intestinal barrier, further highlighting the potential value of mushroom DFs as foods or drugs.

### 5.2. Immunomodulatory Effects

The intestinal tract is the largest immune organ of the human body, and it is involved in immune and inflammatory reactions [153]. Although DFs cannot be completely digested in the intestine, they are decomposed into various metabolites through enzymes produced by intestinal microorganisms. Some microbial metabolites, such as tryptophan metabolites and SCFAs, interact with host cells through the intestinal barrier, thereby affecting the immune response [154]. An increasing number of studies have shown that SCFAs can inhibit the expression of inflammatory factors or alleviate inflammation by promoting histone acetylation or activating GPRs [155], activating peroxisome-proliferator-activated receptors [156], inhibiting the NF-κB signaling pathway [157], facilitating T-cell apoptosis [158], increasing antimicrobial peptide production [159], and downregulating the expression of signal transduction and activating transcription factor-3 [160]. Relevant studies have also reported that DFs from mushrooms can promote SCFA production and the growth of intestinal microorganisms to stimulate the host immune response and regulate the differentiation, maturation, and function of immune cells (Table 3). Vlassopoulou et al. [161] found that supplementation with mushroom β-(1→3, 1→6)-d-glucan is well tolerated and promotes health through the potentiation of the immune system. In addition, *F. velutipes* DF may affect immune function regulation by mediating the gut microbiota [101]. *H. erinaceus* DFs can regulate the gut microbiota composition and immune activity through the NF-κB, MAPK, and PI3K/Akt pathways [127]. *G. lucidum* DFs change the diversity of the gut microbiota and significantly alleviate pancreatitis symptoms in mice by reducing the levels of lipase, interferon-γ (IFN-γ), and TNF-α and increasing SOD levels and total antioxidant activity [124]. *A. auricular* DFs may affect intestinal nutritional metabolism and immune regulation by changing the composition of the gut microbiota [128]. *G. lucidum* DFs not only regulate the gut microbiota composition and SCFA production but also participate in the regulation of gene expression in KEGG pathways related to different types of inflammation [129]. Thus, these studies indicate that DFs from different mushrooms may be associated with different immune regulatory pathways. This immune regulation can be attributed to the diversity of the gut microbiota and SCFA production, which may act as signaling molecules for mediating and maintaining the host’s immune system.

### 5.3. Antitumor Effects

The gut microbiota affects the metabolism and endocrine and immune systems of the host. The gut microbiota is associated with the occurrence of many diseases, including inflammatory bowel disease, nonalcoholic fatty liver disease, type 2 diabetes, and neurodegenerative diseases [162,163,164]. Importantly, increasing evidence shows that the gut microbiota can affect tumor occurrence, tumor progression, and the response to treatment [19]. For example, *Helicobacter pylori* induces gastritis and canceration by producing toxic factors such as cytotoxin-associated gene A and vacuolating cytotoxin A [165]. A study investigating the gut microbiota of patients with early lung cancer reported that *Akkermansia muciniphila* may cause lung cancer [166]. The decrease in the abundance of *Lactobacillidae* and *Bifidobacteriaceae* in colorectal cancer (CRC) patients is related to colon and rectal tumors, respectively [167]. In patients with multiple polypoid adenoma and intramucosal carcinoma, significant changes in the microbiome and metabolome have been observed. The relative abundance of *Fusobacterium nucleatum* spp. increased during the progression of intramucosal carcinoma to the more advanced stage, while the abundance of *Atobobium parvulum* and *Actinomyces odontolyticus* significantly increased in patients with multiple polypoid adenoma and/or intramucosal carcinoma [168]. Moreover, the gut microbiota is closely correlated with the effect of chemotherapy and immunotherapy [169,170]. For example, the gut microbiota can alleviate chemotherapy-induced adverse reactions in CRC patients [171]. Butyric acid, a metabolite of the gut microbiota, can directly improve the antitumor cytotoxic effect of CD8^+^ T cells in vitro and in vivo in an ID2-dependent manner by promoting the IL-12 signaling pathway. Butyric acid can also promote the antitumor efficacy of oxaliplatin [172]. *L. rhamnosus* GG-induced cGAS/STING-dependent type I interferon can enhance the response to immune checkpoint inhibitors [173].

DFs from mushrooms play an anticancer/antitumor role by regulating the gut microbiota composition and diversity, and this role has attracted increasing attention (Table 3). For example, *G. lucidum* polysaccharide can reverse the proportion of *Firmicutes/Bacteroides* and increase the levels of *Alistipes*, resulting in the production of SCFAs, and *Helicobacter* and *Riskenella*, which are related to immunosuppression and carcinogenesis [130]. Moreover, *G. lucidum* DFs can alleviate CRC by altering special intestinal microorganisms [131]. Thus, these studies indicate that mushroom DFs inhibit tumor growth or metastasis by regulating the gut microbiota composition and diversity; furthermore, immunomonitoring mediated by gut microbiota-produced metabolites such as SCFAs may be beneficial. However, the exact anticancer/antitumor mechanism of mushroom DFs remains unclear.

### 5.4. Other Beneficial Effects

Because of their strong water absorption and swelling capacities, DFs from mushrooms, especially IDF, can promote intestinal peristalsis, increase stool volume, increase the frequency of bowel movements, and avert constipation, thereby preventing and treating gastrointestinal diseases. Feeding constipated rats with *A. polytricha* DFs increased the wet weight of their stool and intestinal propulsion rate, thereby indicating high constipation-relieving activity [73]. Furthermore, DFs from mushrooms have antioxidant capacity and can eliminate free radicals from the body. For example, DFs extracted from *Boletus edulis* have significant reducing power and chelating activity and strong antioxidant activity [174].

## 6. Conclusions and Prospects

DFs have various biological activities and are a crucial component of foods that can benefit health. Intestinal microorganisms selectively degrade DFs from mushrooms, thus conferring benefits to the host. The physiological activities of mushroom DFs are usually affected by the extraction method or modified extraction method used and their raw material, such as mycelia, fruiting bodies, and sclerotia. The intake of mushroom DFs induces changes in the host gut microbiota, thereby affecting the host’s immune system. The beneficial effect of DFs on the host may be mediated by gut microbiota-produced metabolites. In particular, SCFAs participate in the regulation of the host’s metabolic homeostasis and immune response. However, until now, the regulatory effect of mushroom DF on the gut microbiota has not been completely investigated. Therefore, future studies on mushroom DFs should aim to (1) investigate the full spectrum of metabolites produced through mushroom DF ingestion and their effect on host immunity by using a combination of multiomics analysis techniques (metagenomics, metabonomics, and other omics); (2) investigate the characteristics of the gut microbiota in different populations and the functional role and mechanisms by which DFs from mushrooms help maintain gut microbiota and host health; and (3) diagnose disease on the basis of specific microorganisms or metabolites and realize the goal of targeted prevention and treatment by using specific DFs. Importantly, additional animal trials and human preclinical and clinical trials are required to fully understand the multiple beneficial effects of DFs from mushrooms on human health using a combination of multiomics analysis techniques.

## Figures and Tables

**Table 1:** Different extraction methods for DF used on different mushroom varieties.

Extraction Methods		Materials	Extraction Conditions	Extraction Features	Reference
Physical method	Pressurized hot water	*Pleurotus sajor-caju*	140 °C, 0.92 MPa, and 40 min	Water as a solvent, low cost, but poor impurity removal	[46]
	Ultrasound-assisted	*Agaricus bisporus*	15 min, 100 mm amplitude, and 1 h of precipitation in 80% ethanol	Less time-consuming and highly efficient, but high cost and little capacity	[47]
	Microwave	*Cordyceps gunnii* mycelia	1:20 (*w*/*v*), 70 °C, 280 W, 5 min	High extraction efficiency, short time and low energy input, but the microwave power and microwave time should be strictly controlled	[48]
Biological method	Enzymatic	*Schizophyllum commune*	α-amylase, 100 °C, 30 min; protease 60 °C, 30 min	High specificity of enzyme is needed, and the extraction conditions must be strictly controlled	[49]
Chemical method	Alkaline	*Coprinus comatus*	2% NaOH in a ratio of 1:15, 85 °C, 2 h	High yield, but may degrade some compounds	[50]
	Acid	*Lentinula edodes* stipe	100 °C, 2 h; 0.8 M trichloroacetic acid, 4 °C, 3 h	High yield, but may produce some byproducts	[51]
Combined method	Hot water and alkaline	*Cookeina tricholoma*	98 °C, 4 h; 2% KOH (*w*/*v* 1:4), 98 °C, 4 h	High yield and purity, but time-consuming	[52]
	Acid–alkaline combined	*Pleurotus eryngii*	0.1 M H_2_SO_4_ (1:10 *w*/*v*), 60 °C, 2 h; 0.25 M NaOH (1:8 *w*/*v*), 60 °C, 2 h	Higher purity, low cost, but may cause excessive degradation	[53]
